# Human health risk assessment of PM_10_-bound heavy metals and PAHs around the Latin America’s Largest opencast coal mine

**DOI:** 10.1007/s11356-023-30787-z

**Published:** 2023-11-27

**Authors:** Heli A. Arregocés, Guillermo J. Bonivento, Luis A. Ladino, Erick Beristain-Montiel, Gloria Restrepo, Javier Miranda, Harry Alvarez-Ospina, Roberto Rojano

**Affiliations:** 1 Grupo de Investigación GISA, Facultad de Ingeniería, Universidad de La Guajira, Riohacha, Colombia; 2 Grupo Procesos Fisicoquímicos Aplicados, Facultad de Ingeniería, Universidad de Antioquia SIU/UdeA, Calle 70 No. 52–21, Medellín, Colombia; 3 Grupo de Investigación ZENTECH, Facultad de Ingeniería, Pontificia Universidad Javeriana, Cra 7 No. 40–62, Bogotá, Colombia; 4 Instituto de Ciencias de La Atmósfera y Cambio Climático, Universidad Nacional Autónoma de México, Mexico City, México; 5 Facultad de Química, Universidad Nacional Autónoma de México, Mexico City, México; 6 Instituto de Física, Universidad Nacional Autónoma de México, Mexico City, México; 7 Facultad de Ciencias, Universidad Nacional Autónoma de México, Mexico City, México

**Keywords:** Inhalation risk, Coal mine, Polycyclic aromatic hydrocarbons, Heavy metals, Cancer risk

## Abstract

Air pollution in opencast coal mine areas is a critical issue, resulting in harmful severe effects on human health. Therefore, it is essential to understand the air pollution factors and to assess the risks to humans. This study evaluated the potential risks (carcinogen and non-carcinogen) of inhalation exposure to PM_10_-bound heavy metals and Polycyclic Aromatic Hydrocarbons (PAHs) in an open pit mine in northern Colombia. During February-May 2022, PM_10_ samples were collected at eight sites. Heavy metals (i.e., Al, Cr(VI), Mn, Cu, Zn, As, Pb) and PAHs (thirteen priority PAHs, and one non-priority PAH) levels linked to PM_10_ were analyzed by X-ray fluorescence and gas chromatography–mass spectrometry, respectively. PM_10_ concentrations were found to range between 4.70 and 59.90 µg m^−3^. Out of the three different zones of the study area (i.e., North Zone, South Zone, and Populated Zone), the North Zone recorded the highest daily average concentrations of Cr(VI) (104.16 ng m^−3^), Mn (28.39 ng m^−3^), Cu (33.75 ng m^−3^), Zn (57.99 ng m^−3^), As (44.92 ng m^−3^), and Pb (27.13 ng m^−3^). The fraction of the analyzed heavy metals at all monitoring sites was 82%–89% for Al, followed by Cr(VI) with 3%–6%. Cr(VI) was the major contributor to the carcinogenic risk values, while Cu, Cr(VI), and As were the main drivers for the non-carcinogenic risk. The average cancer risk range for heavy metals was 3.30 × 10^–04^ –5.47 × 10^–04^. On the other hand, the cancer risk for PAHs exposure was acceptable. The average incremental lifetime cancer risk (ILCR) values varied between 2.87 × 10^–07^ and 4.21 × 10^–07^. Benzo[a]pyrene contributed to 54%–56% of the total risk from inhalation of PM_10_-bound PAHs, while Indeno[1,2,3-cd]pyrene contributed to 16%–19%. Based on the Monte Carlo sensitivity analysis, exposure to Cr(VI) was the main factor affecting cancer risk in the North, South, and Populated Zones. A suitable risk assessment and management plan requires understanding PM_10_-bound heavy metals and PAHs concentration levels as well as their potential health risks, mainly in open-cast coal mine zones. Our study found that people living near open-pit mines face potential health risks, so it is crucial to establish policies and regulations to control emission sources.

**Supplementary Information:**

The online version contains supplementary material available at 10.1007/s11356-023-30787-z.

## Introduction

Around 99% of the world’s population breathe air that exceeds the limits set by the World Health Organization (WHO 2022). Many studies show that air pollution causes significant damage to human health, increasing the risks to the respiratory and cardiovascular systems and aggravating pre-existing diseases (Luo et al. 2020; Tsai et al. 2019; Yang et al. 2022). Air pollution is responsible for more than six million premature deaths worldwide each year (WHO 2022). Open-pit coal mines are associated with negative environmental impacts and adverse effects on public health (Espitia-Pérez et al. 2018). Although the global economic system is aware of a shift towards clean energy systems, coal demand has been essential worldwide in recent years (Nyambuu and Semmler 2020).

Particulate matter (PM) smaller than 10 µm (PM_10_) is the most emitted air pollutant in opencast mining operations (Sharma and Kumar 2023). PM generated from opencast coal mines is a complex mixture of particles that vary in size, morphology, chemical composition, and toxicity (Hao et al. 2018). People exposed to high levels of PM_10_ are more likely to develop chronic respiratory diseases, lung cancer, and cardiovascular disease (Pun et al. 2017; Ziou et al. 2022). The health effects of PM_10_ in mining areas have been related to the chemical composition of the particles, mainly with toxic heavy metals and polycyclic aromatic hydrocarbons (PAHs) (Chen et al. 2022; Marmett et al. 2023; Roy et al. 2019a). PM_10_ containing heavy metals and PAHs can be highly toxic even when the mass concentrations are below the established WHO allowable limits. The International Agency for Research on Cancer (IARC) has classified ambient outdoor PM as a Group 1 human carcinogen. This designation denotes a high degree of evidence supporting the association between exposure to PM and an increased risk of human cancer (Straif et al. 2017).

Some studies have focused on quantifying PM_10_ emissions and their concentration levels to determine the impact of mining activities (Liu et al. 2020). However, the chemical composition of the particles can tell us the types of sources that contribute to high pollution (Dash et al. 2020). Also, PM_10_ chemical characterization, mainly heavy metals, allows us to make inferences concerning health effects (Yadav 2021). The presence of metals linked to PM_10_ can significantly affect health in people living near mining areas (Roy et al. 2019b). Other studies have shown that the Ni and Zn-linked to PM in areas affected by industrial activities can cause mutations and chromosomal abnormalities (Lemos et al. 2012). Additionally, heavy metals like As, Pb, Cd, Fe, Cr, Zn, and Cu have been found to induce micronuclei, chromosomal aberrations, and inhibit mitotic index (Leme and Marin-Morales 2009).

PAHs are present in coal mines due to incomplete combustion of carbonaceous materials. The surrounding areas are also affected by PAHs, primarily caused by vehicular traffic, biomass burning, and coal combustion (Mallah et al. 2022). Out of the different HAPs, benzo[a]pyrene (BaP) and dibenz[a,h]anthracene (DahA) pose the most significant risk due to their carcinogenic properties, which are toxic, mutagenic, and carcinogenic to humans (Morakinyo et al. 2020). PM-bound PAHs have been found to cause chromosome aberrations and affect the distribution of mitosis phases (Cabaravdic 2010). Long-term exposure to some PAHs has been linked to cancer, cataracts, liver and kidney damage, and jaundice (ATSDR 1995; Kim et al. 2013). Additionally, exposure to PAHs may interfere with hormone systems, reduce immune function, and harm reproduction (Garcia-Suastegui et al. 2010). The studies show that the levels of PAHs in coal mines are mainly related to the pyrogenic and petrogenic emission sources and burning activities (Marmett et al. 2023; Suman et al. 2016).

Colombia is the leading coal producer in Latin America (Dominguez et al. 2022). Opencast mining in the northern region of the country is liable for the majority of coal production in Colombia (Aristizabal-H et al. 2023). Mining activities occur in approximately 690 km^2^ (Carmona and Jaramillo 2020). Coal mining activities require drilling and blasting large areas, the use of power shovels, the transport of trucks on unpaved roads, loaders, and the storage of waste material and coal outdoors. Therefore, PM can be directly emitted into the atmosphere by the aforementioned activities. From February to April, PM_10_ levels in northern Colombia have been found to exceeded the allowable limits set by the Colombian government and the WHO (Rojano et al. 2021). Arregocés et al. (2022) indicated that various regional and local factors such as regional atmospheric transport and biomass burning have the potential to impact PM_10_ levels and their composition.

The Colombia's largest indigenous community, Wayuu (380.460 inhabitants) live in La Guajira State, where the coal mine is located (DANE 2019, 2018). Therefore, the Indigenous and Afro-Colombian communities stablished near the coal mine are at high health risk due to exposure to PM_10_ containing heavy metals such as Cr, Cu, and organic compounds such as PAHs (Espitia-Pérez et al. 2018). Morbidity due to respiratory infections in northern Colombia has been higher than the national average by a factor of 2.07 (MinSalud 2018). Additionally, the mortality rate in children ≤ 5 years old due to acute lower respiratory disease has been found to increase with time in recent years (Avilés 2019). The aims of this study are (i) to determine the exposure to PM_10_-bound heavy metals and PAHs and (ii) to assess the human health risk of heavy metals and PAHs bound to PM_10_ via inhalation.

## Materials and methods

### Study area

**Fig. 1:**
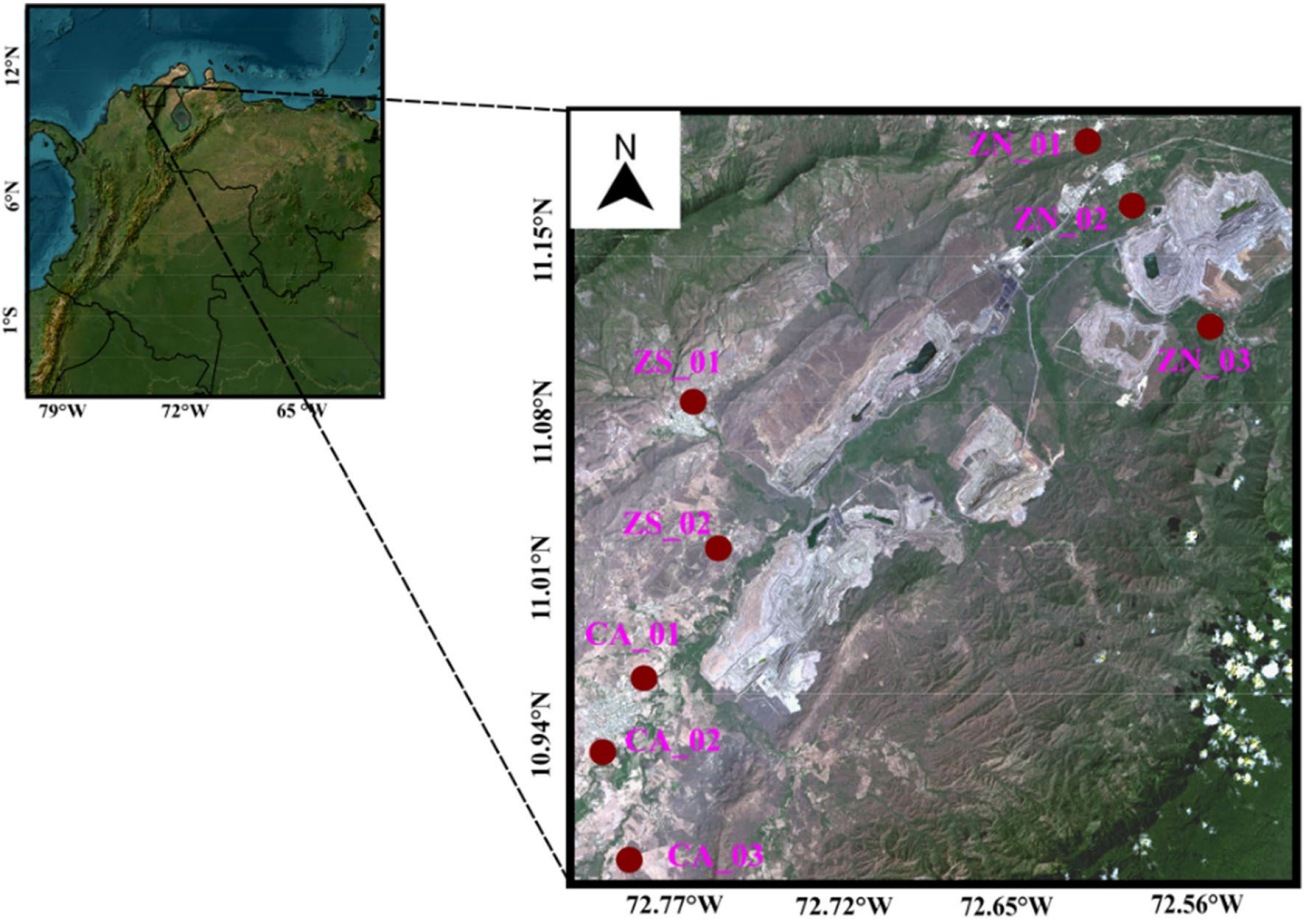
Open-pit coal mine and air quality monitors in the study area: North Zone (ZN_01, ZN_02, ZN_03), South Zone (ZS_01, ZS_02) and Populated Zone (CA_01, CA_02, CA_03)

The largest coal mine in Latin America is located in the northern Colombia (Fig. 1). The coal mine extends over an area of 690 km^2^ in the municipalities of Albania (11°9.6′N, 72°35.4′W), Barrancas (10°57.5′N, 72°47.3′W), and Hatonuevo (11°4.0′ N, 72°45.7′W) in La Guajira State. The opencast coal mine is between the Perijá mountain range to the east and the Santa Marta snow-capped mountain range to the west. The Perijá mountain range is a continuation of the eastern branch of the Andes extending to the Guajira desert in the north. Its peak reaches 3630 m above sea level (ASL). In contrast, the Sierra Nevada is among the highest coastal ranges globally, with an elevation of approximately 5700 m ASL, and is 42 km away from the Caribbean coast. The study domain is classified as a tropical dry forest region whose annual average precipitation is 1200 mm, the annual air temperature ranges between 23 and 32 °C, and the annual average humidity is 71%. February has the highest relative humidity, with an average of 79%, while August has the lowest relative humidity, with an average of 58%.

The region has a human development index (HDI) of 0.68. Approximately 108,000 tons of bituminous coal are mined daily in the region (UPME 2023). The study area is divided in three areas: North Zone, South Zone, and Populated Zone. Most of the people living close to the mine is concentrated in the Populated Zone (≈76% inhabitants). It is important to note that in the study area there are not additional industrial activities, and that the most common economic local activities are goat farming and street food traders. The mining activities use blasting with explosives daily in an area that covers 380 km^2^ whose emits PM_10_ in the mine and its surroundings (Oliveira et al. 2019). Some production pits and dump sites are located approximately two kilometers away from the Indigenous reserves and small rural towns.

### Monitoring of PM_10_

A PM_10_ monitoring campaign was performed at eight sites classified into three groups: North Zone (ZN_01, ZN_02, and ZN_03), South Zone (ZS_01 and ZS_02), and Populated Zone (CA_01, CA_02, and CA_03) as shown in Fig. 1. The PM_10_ monitors were placed upwind of the main urban centers. PM_10_ was collected using PM_10_ High Volume Air Sampler System (Hi-vol) from February to May 2022. Based on the data gathered from PM_10_ monitoring stations, it has been observed that these months have consistently exhibited the highest pollution levels in recent years. The Hi-vols operated at an average standard flow of 1.13 m^3^ min^−1^. The PM_10_ samples were collected on quartz-fiber filters (porosity 0.30 μm) 24 ± 1 h. The PM_10_ concentrations were obtained following the US-EPA CFR Title 40, Part 50, Chapter I, Appendix J Reference Method (EPA 1999) procedure. A total of 210 PM_10_ samples were collected in the eight sampling sites.

Before sample collection, all filter papers underwent preparation in a strictly controlled environment at a temperature range of 22 ± 3 °C and relative humidity of 30 ± 5% for 24 h. The same preparation process was repeated post-sampling. Each filter was subjected to three weights using a microbalance. The difference in filter weight before and after sampling was utilized to determine the mass of the collected particulate matter, which was then divided by the sampled air volume to ascertain its concentration. Monitoring activities were conducted once every three working days using Tisch Environmental sampling equipment (model TE-6070DV-BL), which was purposely designed without a motor brush to prevent the collection of PM chemical elements from the monitoring equipment exhaust.

We obtained meteorological data from a single station located in the ZS_02 site, which provided information on temperature (T), relative humidity (RH), and wind speed (WS). The Weather Research and Forecasting (WRF) model provided data on wind direction (WD) and planetary boundary layer height (PBLH). The WRF model encompasses a mother domain with a spatial resolution of 27 km, located at 11.4°N, 72.5°W, covering the Caribbean Sea. Its main purpose is to capture synoptic features. The first nested domain, with a spatial resolution of 9 km, includes the La Guajira department. The second nested domain, with a spatial resolution of 3 km, covers the west Perijá mountain range and the east Santa Marta snow-capped mountain range. The innermost domain is centered over the open-pit coal mine, consisting of 32 columns and 32 rows of 1 × 1 km2 grid cells. The four domains interact with each other through a one-way nesting strategy, while the model's vertical structure encompasses 60 layers, covering the entire troposphere. In order to validate the WRF simulations, we use data from meteorological stations located at the ZS_02 site (Figure S1).

### PM_10_-bound heavy metals and PAHs

The Al, Cr, Mn, Cu, Zn, As, and Pb concentrations were determined through X-ray fluorescence (XRF). We considered three criteria for metals analysis. (i) heavy metals classified as Group 1 carcinogens and categorized as Group 2A carcinogens by the International Agency for Research on Cancer, (ii) Emission sources in the study area, and (iii) studies conducted and main findings (Arregocés et al. 2020; Espitia-Pérez et al. 2018). The analysis was performed with a custom-built X-ray spectrometer (Espinosa et al. 2012), based on an Oxford Instrument X-ray tube with an Rh anode and silicon drift detector (140 eV resolution at 5.9 keV). The tube was operated at 50 kV and 750 µA, irradiating for 900 s per spectrum. The detection efficiency was determined using a set of thin film standards. The spectra obtained for each sample were decoded using WinQXAS (Margui et al. 2018). The experimental uncertainties for each metal was calculated using the method described by Espinosa et al. (2010). Uncertainties were associated with the thickness of the thin film standards, irradiation time, residual spectral peaks, area of the filters, and volume of air pumped to collect the sample. According to the USEPA model and other studies conducted in coal mines, the concentration of Cr(VI) was determined to be one-seventh of the total concentration of Cr(Espitia-Pérez et al. 2018; Izhar et al. 2016; Roy et al. 2019b; Wang et al. 2023).

The geo-accumulation index (${I}_{geo}$) was used to assess the levels of heavy metals pollution in ambient air. The ${I}_{geo}$ values are determined for each PM_10_-bound heavy metal as established by Muller (1969). The ${I}_{geo}$ for each heavy metal is calculated by taking the logarithm (base 2) of the metal's concentration and dividing it by an adjustment factor (1.50) multiplied by the metal's background value in the soil. The soil samples' background values were obtained from previous studies in Colombia (Marrugo-Negrete et al. 2017; Pallares et al. 2022). The geo-accumulation index values indicate pollution levels as follows: No pollution (${I}_{geo}$ ≤ 0), Unpolluted to moderate pollution (0 < ${I}_{geo}$  ≤ 1), Moderate pollution (1 < ${I}_{geo}$  ≤ 2), Moderate to heavy pollution (2 < ${I}_{geo}$  ≤ 3), Heavy pollution (3 < ${I}_{geo}$  ≤ 4), Heavy to extreme pollution (4 < ${I}_{geo}$  ≤ 5), and Extreme pollution (${I}_{geo}$ > 5). Al-${I}_{geo}$ value was not determined due to the absence of background soil values in the region.

On the other hand, the variability of 13 priority PAHs, according to the US-EPA (i.e., Naphthalene (Nap), Acenaphthylene (Acy), Acenaphthene (Ace), Fluorene (Flu), Phenanthrene (Phe), Anthracene (Ant), Fluoranthene (FR), Pyrene (Pyr), Benz[a]anthracene (BaA), Benzo[k]fluoranthene (BkF), Indeno[1,2,3-cd]pyrene (IDP), Benzo[b]fluoranthene (BbF), and Benzo[a]pyrene (BaP)) and one Non-priority PAH (i.e., Triphenylene (Tri)) were analyzed. Benzo[ghi]perylene, chrysene, and dibenz[a,h]anthracene were not included in this study because their concentrations were below the detection limits of the used setup. The PAHs extraction from the PM_10_ samples was based on ultrasound-assisted extraction. First, we extracted samples from the 4.7 cm diameter filter in a 5 mL dichloromethane solution (JT Baker, USA). Subsequently, the extract was decanted into a conical tube after the solution placed three times an ultrasonic bath (Branson, 40 kHz, 360 W) for 5 min at 60 °C. The resulting extracts were placed in a gentle stream of N until up less than 1 g was reached. Afterward, the sample was spiked with 250 ng of phenanthrene-D10 as an internal standard to the concentrated extract and the mass was adjusted to 1.0 g before coupling to gas chromatography–mass spectrometry (GC–MS) analysis.

PAHs were determined using a gas chromatograph coupled to a mass spectrometer (Agilent 7890B/5977A) (Bouchonnet 2013). For this purpose, we inject 1 µL of the sample solution at 250 °C in a multi-mode injector operated in splitless mode (1 min). The flow rate was 1.0 mL min^−1^ using helium (99.99%, Infra, Mexico) as carrier gas. The column used was a DB-35MS (35% polyphenylsiloxane) of 30 m × 0.25 mm × 250 µm. The temperature program was as follows: Initial temperature of 65 °C hold for 1 min, after ramp rate of 15 °C/min to 130 °C; followed by ramp rate of 20 °C/min to 210 °C; the following sequence was ramp rate of 2 °C/min, 5 °C/min, and 20 °C/min to 220 °C, 250 °C, and 320 °C, respectively; final temperature 320 °C hold for 4 min. The transfer line temperature was set to 320 °C. The mass spectrometer was operated with electron ionization at 70 eV, using the scan mode from 50 to 550 Da. The identification was carried out with four ions for each compound, and the most abundant ion served as a quantifier ion. The temperature of the ionization source was 230 °C and the quadrupole 150 °C. The total analysis time was 30.5 min.

### Quality assurance and quality control (QA/QC)

In order to adjust the Hi-Vol volume, we utilized the TE-5028 Calibration Kit and TE-HVC-V Xcalibrator HiVol Calibrator. Furthermore, we measured the filter papers' weight differences before and after sampling by using a five-digit microbalance to determine the PM_10_ concentration level. We ensured the microbalances were used and operated correctly by conducting a thorough check. To guarantee proper QA/QC, we checked for accuracy in total sampling times, average actual volumetric flow rates, and gross filter weights. Additionally, we performed a "standard weight" check at the start of each weighing session and carried out calibration checks after every 12 filters were weighed.

To determine PM10-bound heavy metals to ensure the accuracy of our XRF system, we analyzed two thin film standards (Al and Fe) daily and found differences below 1% in the Kα X-ray peaks of both elements. We conducted replicate analyses on all samples and calculated combined uncertainties for each sample. These uncertainties ranged from 7% for major elements to 14% for less abundant ones (Table S1). We calibrated the X-ray detection system with thin film standards, irradiating for 300 s under previous conditions. Furthermore, we evaluated accuracy with the National Institute of Standards and Technology (Gaithersburg, MA, USA) Standard Reference Material SRM-2783 (Air particulate on filter media).

We utilized the standard reference material SRM 1649a from the National Institute of Standards and Technology (NIST) to evaluate the presence of PAHs (Gaithersburg MD, USA) (Amador-Muñoz et al. 2008). We extracted 74% of the organic matter from the sample using solvents. The PAH recovery efficiencies ranged from 61% (Indeno[1,2,3-cd]pyrene) to 87% (Fluorene) when 100 mg of urban dust were extracted (Table S2). The PAH values in the samples were adjusted by subtracting the low PAH amount in the filter field blanks and adjusting those values with their respective recoveries based on the amount of urban dust extracted (between 5 and 150 mg). Subsequently, we selected ion masses based on the mass spectral base peak.

### Health risk assessment

The health risk assessment for PM_10_-bound heavy metals was performed according to Eqs. 1–4 (EPA 2011), while for PM_10_-bound PAHs, Eqs. 5 and 6 were used (EPA 2005; OEHHA 2005). The values used for the variables comprising each equation are shown in Table S3.

1 $${EC}_{i}=\frac{{C}_{i}\times ET\times EF\times ED}{{AT}_{n}}$$

2 $$HQ=\frac{{EC}_{i}}{{RfC}_{i} \times CF}$$

3 $$HI=\sum_{1}^{n}{HQ}_{i}$$

4 $$CR={EC}_{i}\times IUR$$

5 $${TEQ}_{total}=\sum {TEQ}_{i}=\sum {(C}_{i}\times {TEF}_{i})$$

6 $$ILCR=\frac{{TEQ}_{total}\times SF\times IR\times EF\times ED\times {10}^{-06}}{BW\times AT}$$

where ${C}_{i}$ represents the heavy metal concentrations (µg m^−3^) or PAHs concentrations (ng m^−3^), ${EC}_{i}$ is the daily intake attributed to exposure to chemical concentrations via inhalation (µg m^−3^), $EF$ is the exposure frequency (Days years^−1^), $ED$ is the exposure duration (year), $ET$ is the exposure time (h day^−1^), $IR$ is the inhalation rate (m^3^ day^−1^), ${AT}_{n}$ is the average lifetime (hours), ${RfC}_{i}$ is the chronic inhalation reference concentrations (mg m^−3^), $SF$ is the inhalation carcinogenic slope factor (mg kg^−1^ day^−1^), AT is the average lifespan (days), BW is the body weight (kg), and CF is a conversion factor (μg mg^−1^). $HI$ is the hazard index due to exposure to heavy metals via inhalation. We used a hazard quotient ($HQ$) to assess the risks of non-cancerous health effects. If $HQ$ for a metal component is above 1, there could be potential exposure risks. On the other hand, an $HQ$ value below 1 indicates that there should be no adverse health outcomes from exposure to heavy metals. If $HI$ is greater than 1, non-carcinogenic risks from exposure to a mixture of metals are possible.

On the other hand, to determine the cancer risks due to exposure to heavy metals via inhalation, $CR$ (lifetime excess cancer risk) values are evaluated. $CR$ values below 1 × 10^–06^ are considered a negligible risk, 1 × 10^–06^ < $CR$< 1 × 10^–04^ are considered acceptable, and $CR$ > 1 × 10^–04^ is likely to be harmful to humans (Wu et al. 2019). The *CR* value shows the change in developing any cancer by an individual owing to lifetime exposure to metals. Cancer risks via inhalation of Al, Zn, Cu, and Mn are not discussed because inhalation unit risk (IURs) for these metals are unavailable. In PAHs risk assessment, ${TEQ}_{total}$ represents the sum of toxicity equivalence concentrations for all PAHs calculated through the toxic equivalent factor of individual PAHs (${TEF}_{i}$, ng m^−3^). $ILCR$ is the incremental lifetime cancer risk from ambient exposure to PM_10_-bound PAHs. $ILCR$ < 1 × 10^–06^ is considered an acceptable carcinogenic risk value, while $ILCR$ >1 × 10^–4^ are considered high probabilities of lifetime cancer risks from inhalation of PM_10_- bound PAHs (EPA 2011; US EPA 1989). We used the Shapiro–Wilk test to assess the normality of exposure concentration, *HQ*, and *CR* values (Villasenor Alva and Estrada 2009). Mann–Whitney U test was applied to assess the differences between exposure concentrations and the chronic daily inhalation intake for each chemical component analyzed (MacFarland et al. 2016).

We utilized the conditional probability function (CPF) method, outlined in Uria-Tellaetxe and Carslaw (2014), to identify source contributions and risk areas related to PM_10_-bound heavy metals and PAHs exposure. We computed the HI, CR, and ILCR for each zone to determine the spatial distribution of non-cancer risks from PM_10_-bound heavy metals, the cancer risk from exposure to heavy metals, and incremental lifetime cancer risk from ambient exposure to PM_10_-bound PAHs. We coupled CPF with the forward-back-trajectory cluster analysis to provide further insight into the contributions of sources and risk areas. The study area was divided into equally sized grids ($i\times j$). The CPF method calculated the number of air mass trajectories passing and going over each grid at 12-h intervals, estimating the probability of the occurrence of HI, CR, and ILCR values. When a grid cell ($ij$) contained very few trajectories, most of which were associated with high HI, CR, and ILCR values at the receptor site, it was not statistically valid due to the uncertainty related to the small number of trajectories (Kim et al. 2016). Therefore, a weighting function was used to decrease the effects on the CPF calculation. The CPF analysis identified potential risk areas in geographic regions without distributing the contribution of the identified risk to the estimated receptor data. The $ij$ cells identified by the CPF analysis indicated areas with a higher probability of contributing to the calculated risk at the monitoring site.

### Uncertainty and sensitivity analysis

We used a Monte Carlo simulation to analyze the cancer risk associated with each inhalation exposure, with a 95% confidence level. We utilized a probabilistic uncertainty analysis technique that assigned a probability density function to each input parameter to achieve this. We then selected random values from each distribution and input them into the simulation's exposure equations, essential components. This process determined the distribution of predicted values and the sensitivity of the significant input parameters. The Monte Carlo algorithm is a method used to estimate the stochastic properties of human exposure and uncertainty through probability. The Monte Carlo simulation was adapted to minimize uncertainties and quantify risk levels. In this study, we run 10000 iterations and randomly select a value for each variable parameter from its respective distribution. Uncertainty can significantly impact the results of specific studies, like health risk assessments. This uncertainty is often caused by incorrect input parameter usage. Thereby the Monte Carlo simulation technique can be utilized to reduce uncertainty. Following EPA recommendations, exposure parameters such as EF, ED, and ET were assigned values. Inhalation rate and Body weight parameters were analyzed for sensitivity during the study.

## Results

### PM_10_ concentrations

The daily average PM_10_ concentrations were found to be 23.09 ± 10.31 µg m^−3^, 26.46 ± 9.02 µg m^−3^, and 25.11 ± 11.47 µg m^−3^ for the North Zone, South Zone, and Populated Zone, respectively. The highest monthly average PM_10_ concentration were recorded in May (28.68 µg m^−3^), while March reported the lowest monthly average PM_10_ concentrations (< 23.44 µg m^−3^) over the three zones (Table 1). Changes in WS and the PBLH are known to affect the PM_10_ concentration (Banks and Baldasano 2016). The lowest PBLH was observed in May with daily average values between 529.07 m–586.07 m AGL (Table 1). Table 1 shows that an average daily WS above 5.60 m s^−1^ and a daily PBLH between 770 and 863 m AGL were estimated for March.

**Table 1:** Statistical summary of PM_10_ concentrations and meteorology variables during February-May 2022 in the study area. The meteorological variables were determined by the WRF model (Table S4)

		PM_10_ (µg m^−3^) ± stdev	Max–Min (µg m^−3^)	T (°C)	RH (%)	WS (m s^−1^)	WD (°)	PBLH (m AGL)
North Zone	Feb	19.93 ± 9.47	44.94–7.75	27.39	77.95	3.73	97.47	570.7
	Mar	17.98 ± 6.00	30.91–4.70	26.88	74.93	5.61	73.09	770.54
	Apr	28.08 ± 11.88	59.91–8.01	27.16	77.72	4.98	99.23	681.97
	May	28.68 ± 8.24	43.12–15.20	27.68	78.4	4.39	96.39	586.07
	**x̄**	**23.09 ± 10.32**	**59.98–4.76**	**27.28**	**77.25**	**4.68**	**91.55**	**652.32**
South Zone	Feb	28.34 ± 6.46	39.62–18.63	27.21	76.3	3.62	82.36	515.24
	Mar	23.10 ± 9.08	31.51–13.60	27.13	71.57	6.63	41.58	846.97
	Apr	26.46 ± 8.02	39.50–16.80	27.13	76.17	5.34	71.01	679.98
	May	28.61 ± 10.99	46.62–19.73	27.52	77.12	3.85	84.8	529.07
	**x̄**	**26.47 ± 9.02**	**46.65–13.66**	**27.25**	**75.29**	**4.86**	**69.94**	**642.81**
Populated Zone	Feb	26.06 ± 9.26	42.21–15.80	27.18	76.44	3.26	100.09	556.65
	Mar	23.43 ± 9.92	35.20–13.22	27.2	70.92	6.47	29.73	862.12
	Apr	25.11 ± 12.77	43.23–13.12	27.08	76.27	4.91	67.45	666.8
	May	27.59 ± 13.84	46.73–16.10	27.48	77.1	3.51	89.74	540.77
	**x̄**	**25.11 ± 11.47**	**46.77–13.14**	**27.24**	**75.18**	**4.54**	**71.75**	**656.59**

### PM_10_-bound heavy metals and PAHs

**Fig. 2:**
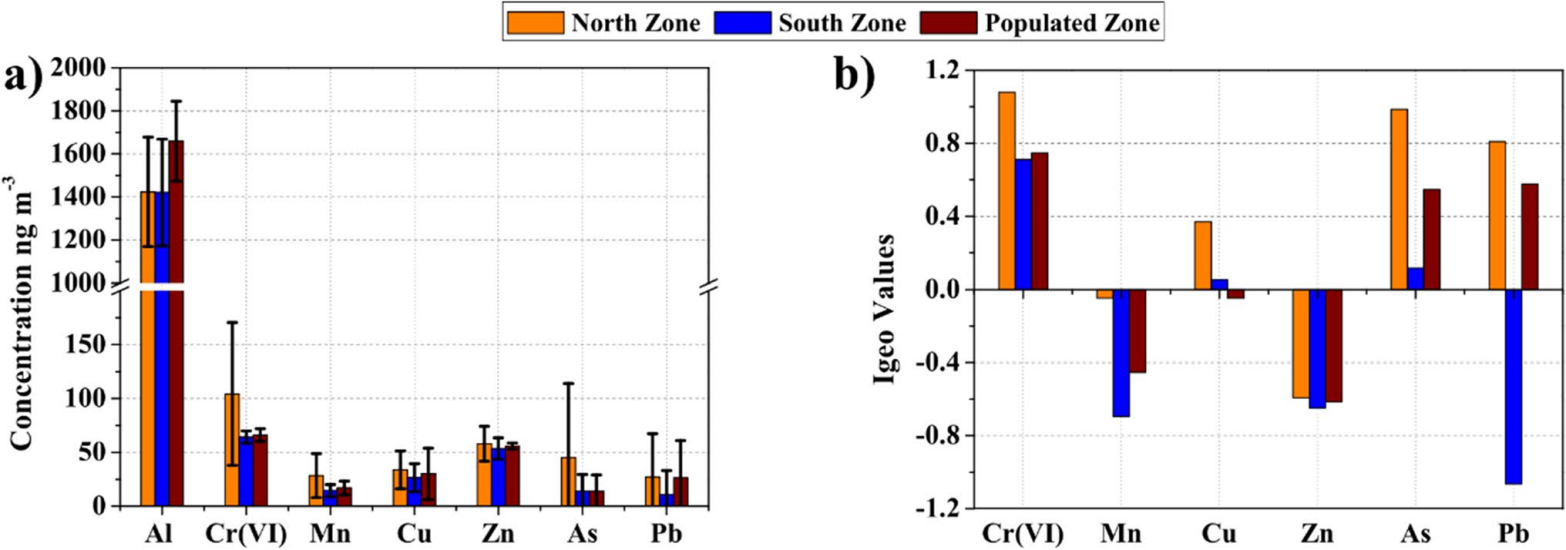
Heavy metals in PM_10_ samples around the largest open-pit coal mine in Latin America (**a**) Average concentrations of PM_10_-bound heavy metals and (**b**) Geoaccumulation index values

The statistical summary of the PM_10_-bound heavy metals level is shown in Table 2. The average concentrations of PM_10_-bound heavy metals, ordered by the highest to the lowest concentration, were Al > Cr(VI) > Zn > As > Cu > Mn > Pb for the North Zone, Al > Cr(VI) > Zn > Cu > Mn > As > Pb for the South Zone, and Al > Cr(VI) > Zn > Cu > Pb > Mn > As for the Populated Zone. As shown in Fig. 2, the highest daily average concentrations of Cr(VI) (104.16 ng m^−3^), Mn (28.39 ng m^−3^), Cu (33.75 ng m^−3^), Zn (57.99 ng m^−3^), As (44.92 ng m^−3^), and Pb (27.13 ng m^−3^) were recorded in the North Zone. On the other hand, higher values of Al (1658.94 ng m^−3^) were recorded in the Populated Zone (Fig. 2a).

**Table 2:** Average, maximum, and minimum concentrations of PM_10_-bound heavy metals (ng m^−3^) for each zone

	North Zone			South Zone			Populated Zone		
	Mean ± stdev	Max	Min	Mean ± stdev	Max	Min	Mean ± stdev	Max	Min
Al	1423.40 ± 254.60	1938.61	1074.84	1420.90 ± 248.11	1790.71	1051.70	1658.94 ± 185.82	2017.00	1316.31
Cr(VI)	104.16 ± 66.28	581.90	57.51	64.08 ± 5.74	75.69	55.77	66.03 ± 5.78	77.03	51.90
Mn	28.39 ± 20.4	163.84	9.55	14.56 ± 5.57	23.29	4.77	16.9 ± 6.35	31.90	4.50
Cu	33.75 ± 17.52	123.84	10.91	26.48 ± 13.12	73.16	10.30	30.07 ± 23.83	152.44	4.01
Zn	57.99 ± 16.31	129.37	32.53	53.6 ± 9.97	69.11	38.12	55.85 ± 12.87	112.16	34.72
As	44.92 ± 68.9	510.12	BLD*	13.99 ± 15.42	42.09	BLD	13.87 ± 15.08	49.57	BLD
Pb	27.13 ± 40.25	131.68	BLD	10.97 ± 22.14	58.43	BLD	26.23 ± 34.71	94.13	BLD

*BLD: Below Limit of Detection

The proportion of heavy metals analyzed shows that 82%–89% corresponds to Al, followed by Cr(VI) with 3%–6% ratios. The lowest proportions correspond to the presence of Pb and Mn, with 0.7%–1.6% and 0.9%–1.7%, respectively. The correlation of Cu and Zn was 0.56 ± 0.32, while the Cu/Zn ratio was 0.66 ± 0.29 in all monitoring sites. The US-EPA sets the limit for airborne Cr(VI) at 12 ng m^−3^ (USEPA 1999); however, all monitoring sites exceeded this value by a factor of 5.5– 8.7 in the three zones.

The *I*_*geo*_ values for Cr(VI), Mn, Cu, Zn, As, and Pb in the study domain can be visualized in Fig. 2b. The *I*_*geo*_ values for Cr(VI) and As were higher than 0 in all three classified zones. In the North Zone, the *I*_*geo*_ of Cr(VI) (1.08) and As (0.99) are moderate. In the other two zones, these elements are present in the atmosphere at the *I*_*geo*_ ranging from unpolluted to moderately polluted. The *I*_*geo*_ values for Pb were estimated to be 0.81 and 0.58 in the North Zone and Populated Zones, respectively. The *I*_*geo*_ calculation for Zn and Mn is below 0, putting these elements in the class of practically uncontaminated.

**Fig. 3:**
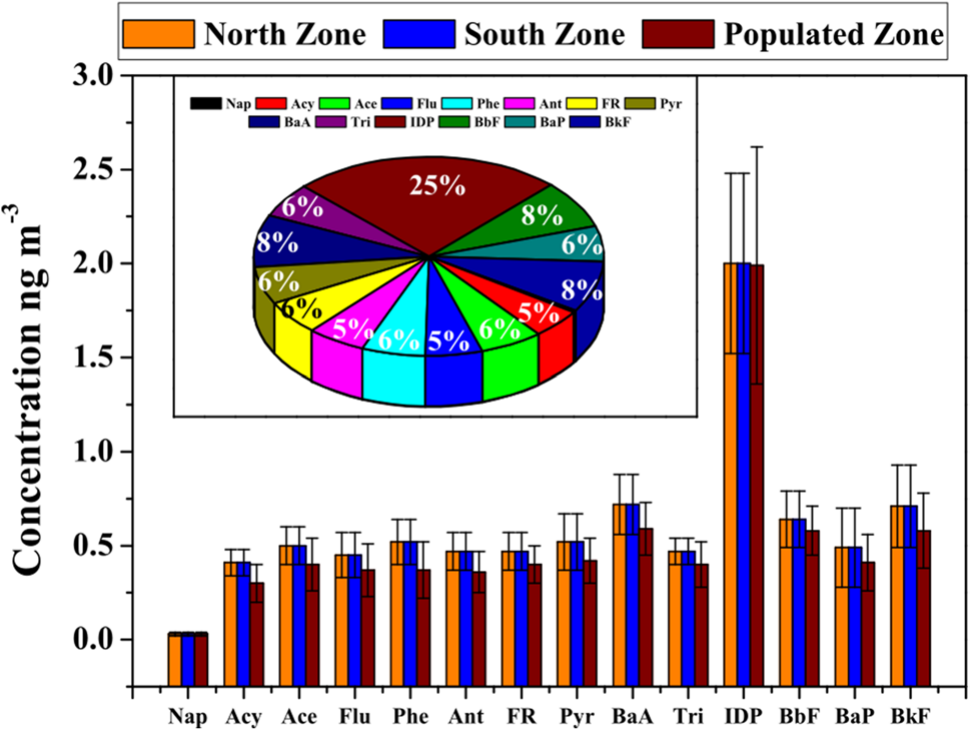
PM_10_-bound PAHs samples around the largest open-pit coal mine in Latin America

The average concentrations of all PAHs were 8.39 ± 0.15 ng m^−3^, 10.40 ± 0.17 ng m^−3^, and 7.21 ± 0.16 ng m^−3^ for the North Zone, South Zone, and Populated Zone, respectively. The average PM_10_-bound PAHs concentrations together to maximum-minimum values in the three zones are shown in Table 3. Out of the 14 HAPs, IDP was found as the most abundant with a fraction corresponding to 24%–28%, followed by BaA with 8%–9%. The South Zone recorded the highest daily concentrations of IDP and BaA, with values of 2.83 ± 0.63 ng m^−3^ and 0.91 ± 0.20 ng m^−3^, respectively. The mean concentration for all the PAHs were lower in Populated Zone compared to values observed in other zones (Fig. 3). Daily average BaP concentrations were found to range between 0.41 and 0.60 ng m^−3^ with a maximum value of 0.92 ng m^−3^.

**Table 3:** Mean, standard deviation, maxima, and minima of PM_10_-bound PAHs (ng m^−3^) from February to May 2022 at the largest open-pit coal mine in Latin America

	North Zone			South Zone			Populated Zone		
	Mean ± stdev	Max	Min	Mean ± stdev	Max	Min	Mean ± stdev	Max	Min
Nap	0.03 ± 0.01	0.06	0.01	0.04 ± 0.01	0.06	0.03	0.03 ± 0.01	0.06	0.01
Acy	0.41 ± 0.07	0.59	0.10	0.44 ± 0.06	0.6	0.35	0.30 ± 0.10	0.63	0.10
Ace	0.50 ± 0.10	0.83	0.13	0.62 ± 0.09	0.78	0.47	0.40 ± 0.14	0.82	0.12
Flu	0.45 ± 0.12	0.8	0.14	0.54 ± 0.14	0.71	0.36	0.37 ± 0.14	0.76	0.13
Phe	0.52 ± 0.12	0.91	0.14	0.64 ± 0.09	0.81	0.49	0.37 ± 0.15	0.84	0.12
Ant	0.47 ± 0.10	0.78	0.16	0.49 ± 0.08	0.70	0.37	0.36 ± 0.11	0.85	0.12
FR	0.47 ± 0.10	0.72	0.17	0.64 ± 0.15	0.81	0.40	0.40 ± 0.10	0.77	0.14
Pyr	0.52 ± 0.15	0.92	0.18	0.63 ± 0.13	0.80	0.38	0.42 ± 0.12	0.82	0.15
BaA	0.72 ± 0.16	1.15	0.21	0.91 ± 0.20	1.17	0.61	0.59 ± 0.14	1.28	0.21
Tri	0.47 ± 0.07	0.69	0.22	0.48 ± 0.13	0.66	0.28	0.40 ± 0.12	0.66	0.19
IDP	2.00 ± 0.48	3.61	0.7	2.83 ± 0.63	3.67	1.80	1.99 ± 0.63	3.69	0.90
BbF	0.64 ± 0.15	1.12	0.21	0.85 ± 0.26	1.23	0.49	0.58 ± 0.13	1.24	0.24
BaP	0.49 ± 0.21	0.92	0.20	0.60 ± 0.17	0.83	0.28	0.41 ± 0.15	0.92	0.17
BkF	0.71 ± 0.22	1.18	0.33	0.69 ± 0.23	0.99	0.31	0.58 ± 0.20	1.06	0.28

Pie charts representing daily mean concentrations of PAHs component in PM10-bound PAHs samples at domain study.

**Fig. 4:**
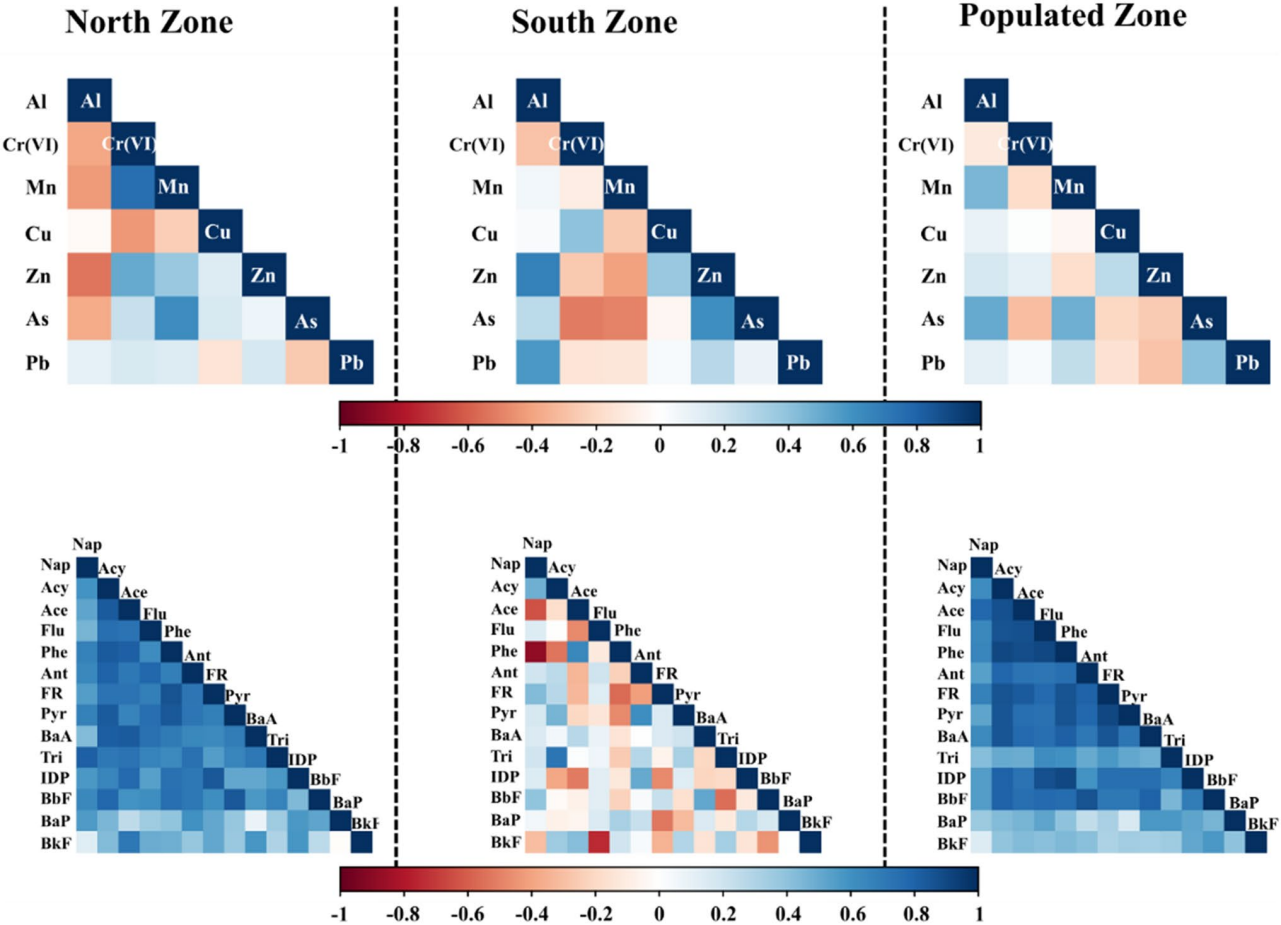
Correlation matrix for PM_10_-bound heavy metals and PAHs at North Zone, South Zone, and Populated Zone

Figure 4 presents the targeted heavy metals and PAHs' Pearson correlation (r) matrix. A moderate positive correlation between Mn and Cr(VI) and As with Zn (r = 0.4–0.8; p < 0.05) was found in the North Zone. Al has a negative correlation with Cr(VI), Mn, Zn, and As. On the other hand, Al and Zn positively correlated with As and Pb in the South Zone. Al was found to be positively correlated with Mn and Zn in the Populated Zone. Simultaneously lightly negative correlation between As with Cr(VI) and Pb with Zn was found respectively. A notable correlation between PAHs was identified within the North Zone and Populated Zone. Specifically, Acy and Ace, Flu, Phe, FR, Pyr, BaA, IDP, BbF exhibited significant positive correlations (r =  > 0.60; p < 0.05). Furthermore, Ace showed a notably strong correlation (> 0.80) with Phe, Phe was strongly correlated with FR and Pyr, and Pyr was highly associated with BbF. In contrast, Nap demonstrated a negative correlation with Phe, while Flu exhibited a negative correlation with BkF within the Populated Zone.

The Ant/(Ant + Phe) ratios obtained in this study were 0.47 ± 0.13, 0.55 ± 0.14, and 0.49 ± 0.11 for the North Zone, South Zone, and Populated Zone, respectively. The overall Ant/(Ant + Phe) ratio range was found to go from 0.88 ± 0.03 to 0.91 ± 0.04. On the other hand, the ratios of FR/(FR + Pyr) obtained were above 0.90 (Table S5).

### Non-cancer risk and Cancer risk

**Fig. 5:**
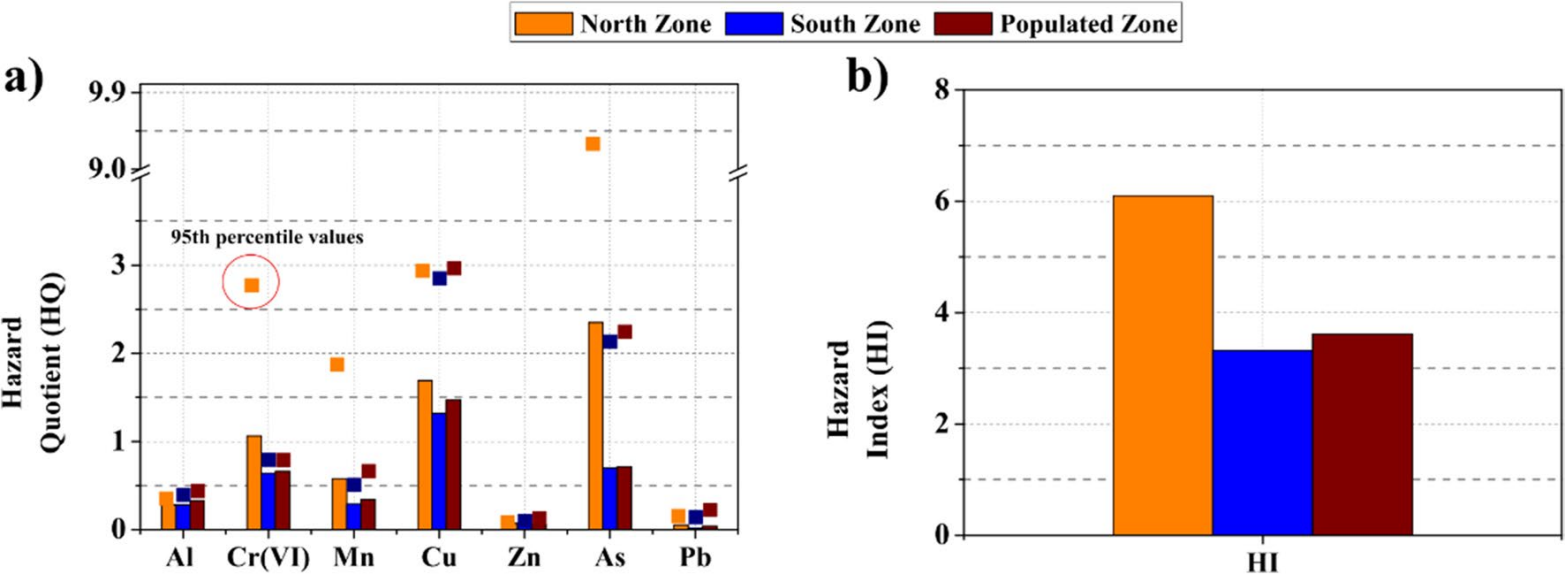
Non-cancer risks of PM_10_-bound heavy metals via inhalation in three zones at the opencast coal mine

Non-cancer risks of PM_10_-bound heavy metals via inhalation in the three zones of the open-pit coal mine are shown in Fig. 5. Cu has the highest *HQ* value of 1.32 and 1.47 for the South Zone and Populated Zone, respectively, while As has the highest *HQ* value of 2.35 in the North Zone. In addition, Cr(VI) and As values are higher than 1.0 in the North Zone. Pb has the lowest *HQ* value for all three zones (0.02 –0.04). *HI* values were above considered safe values (1.0) with averages of 6.10, 3.32, and 3.61 for the North Zone, South Zone, and Populated Zone, respectively, indicating that heavy metals in PM_10_ may pose non-cancer risks for the population. The average *HI* value in the North Zone is higher by a factor of 0.84 and 0.69 times compared to the South and Populated Zone.

Table 4 shows the *CR* values of PM_10_-bound heavy metals and ILCR of PM_10_-PAHs via inhalation in the three zones at the opencast coal mine. The *CR* for Cr(VI) exposure was the highest value in all three zones. The average (95th percentile values) of *CR* for the Cr(VI) was 5.47 × 10^–04^ (1.41 × 10^–03^), 3.30 × 10^–04^ (3.75 × 10^–03^), and 3.38 × 10^–04^ (3.88 × 10^–03^) for the North Zone, South Zone, and Populated Zone, respectively. All values exceeded the acceptable risk limits. Therefore, it can be inferred that five out of the ten thousand inhabitants living in the North Zone may develop cancer due to Cr(VI) inhalation during their lifetime. The mean values of As (2.58 × 10^–05^ –8.68 × 10^–05^) and Pb (5.64 × 10^–08^ –1.37 × 10^–07^) are considered within the acceptable risk ranges. Our analysis utilizing Monte Carlo sensitivity indicates that exposure to Cr(VI) significantly impacts cancer risk in the North Zone, South Zone, and Populated Zone. Furthermore, we have identified specific components associated with coal and gasoline combustion from vehicles and emissions from domestic waste incineration. These findings suggest that open-pit coal mining operations significantly impact cancer risk levels in the study area (Figure S2).

**Table 4:** Cancer risk (*CR*) of PM_10_-bound heavy metals and the ILCR of PM_10_-PAHs via inhalation in three zones at the opencast coal mine

	North Zone			South Zone			Populated Zone		
	Mean	5th	95th	Mean	5th	95th	Mean	5th	95th
Cr(VI)	5.47 × 10^–04^	3.03 × 10^–04^	1.41 × 10^–03^	3.30 × 10^–04^	2.94 × 10^–04^	3.75 × 10^–04^	3.38 × 10^–04^	2.84 × 10^–04^	3.88 × 10^–04^
As	8.68 × 10^–05^	-	3.52 × 10^–04^	2.58 × 10^–05^	-	7.35 × 10^–05^	2.61 × 10^–05^	-	8.26 × 10^–05^
Pb	1.37 × 10^–07^	-	5.23 × 10^–07^	5.64 × 10^–08^	-	2.95 × 10^–07^	9.49 × 10^–08^	-	4.37 × 10^–07^
PAHs (ILCR)	3.29 × 10^–07^	1.59 × 10^–07^	5.21 × 10^–07^	4.21 × 10^–07^	3.10 × 10^–07^	4.98 × 10^–07^	2.87 × 10^–07^	1.64 × 10^–07^	5.13 × 10^–07^

The average *ILCR*, in descending order, was 4.21 × 10^–07^, 3.29 × 10^–07^, and 2.87 × 10^–07^ for the South Zone, North Zone, and Populated Zone, respectively (Table 4). This suggests that the *ILCR* from exposure and inhalation of PAHs are acceptable; even if we consider the 95th percentile values, as the acceptability range remains within the established values. BaP contributes 54% –56% of the total inhalation risk of PM_10_-bound PAHs, while IDP contributes 16%–19%. After analyzing the percentage contribution and Monte Carlo sensitivity output, PM10-bound PAHs do not pose a significant cancer risk in the area under study. However, our study of PAHs highlights that IDP and BaA are the most noteworthy contributors to cancer risk levels in the open pit coal mine and nearby cities (Figure S2).

### Geographical risk distribution

**Fig. 6:**
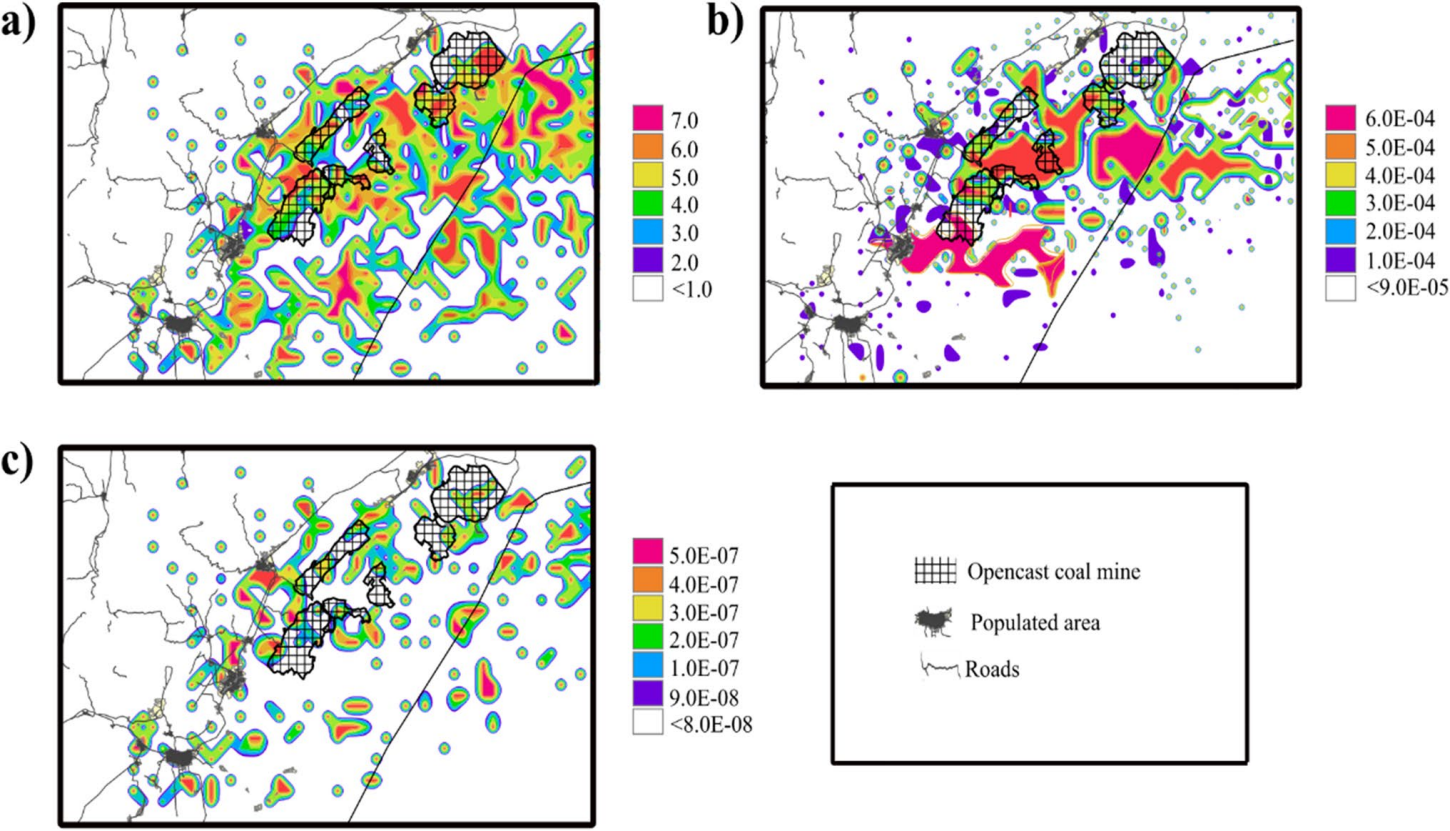
Risk areas due to the exposure of PM_10_-bound heavy metals and PAHs integrating a conditional probability function for estimated HI, CR, and ILCR. (**a**) Spatial potential non-cancer risk distributions of PM10-bound heavy metals (**b**) Spatial cancer risk distributions due to exposure to heavy metals, and (**c**) Spatial potential incremental lifetime cancer risk distributions from ambient exposure to PM_10_-bound PAHs

Figure 6 presents each zone's HI, CR, and ILCR computed. After conducting a CPF analysis, two potential risk areas were identified. The northeast direction from nearby sources between the North and South Zone pits showed the highest risk contributions, with estimated non-cancer risks of PM_10_-bound heavy metals ranging from 3.32 to 6.10. Estimated inhalation of Cr(VI), As, and Pb in these areas resulted in CR daily values reaching 6.33 × 10^–04^. Another potential risk area was identified in the west-southwest directions of the South Zone, with HI and CR values of < 5 and 1.00 × 10^–04^, respectively. These areas pose a risk of non-cancer and cancer due to exposure to heavy metals. The west-southwest directions of coal mining pits in the South Zone showed higher potential incremental lifetime cancer risk values due to PM_10_-bound PAHs, with values up to 5 × 10^–07^.

## Discussion

This study analyzed the potential risks of inhalation exposure to PM_10_-bound heavy metals and PAHs in an open pit mine in northern Colombia. The study found that the North Zone recorded the highest daily average concentrations of heavy metals, with Cr(VI) being the major contributor to the carcinogenic risk values. Cu, Cr(VI), and As were the main drivers for the non-carcinogenic risk. The cancer risk for PAH exposure was acceptable. Overall, exposure to Cr(VI) was the main factor affecting cancer risk in all three zones.

The monthly average PM_10_ concentration in May (28.68 µg m^−3^) was the highest, while March had the lowest (< 23.44 µg m^−3^) across the three zones (Table 1). A decrease in the diurnal PBLH cycle have been associated with episodes and increased surface PM concentration (Yin et al. 2019), therefore, it is very likely that this low PBLH is responsible for the high PM_**10**_ observed in May. On the other hand, several favorable conditions that reduced PM_10_ concentrations were observed in March. Generally, intense WS (> 5.60 m s^−1^) disperse PM_10_ from areas close to emission sources. Furthermore, deeper PBLH increases the vertical pollutant dispersion and the ventilation coefficient (Lu et al. 2023).

The proportion of heavy metals analyzed shows that 82%–89% corresponds to Al, followed by Cr(VI) with 3%–6% ratios. The presence of Pb and Mn were 0.7%-1.6% and 0.9%-1.7%, respectively (Table 2). The Al content in PM_10_ is related to soil erosion and resuspended road dust (Tiittanen et al. 1999). Additionally, atmospheric Al has been linked to the release of coal combustion and domestic waste incineration emissions (Alasfar and Isaifan 2021). Cu, Zn, and Pb are considered tracers of industrial and vehicular emissions and coal combustion (Das et al. 2015). The correlations (0.56 ± 0.32) and rations (0.66 ± 0.29) of Cu and Zn suggest the presence of vehicular emission sources. Atmospheric Pb is related to vehicle emissions (Hao et al. 2018). The presence of Pb can be attributed to haul trucks up to 320 Ton capacity and vehicles that travel on unpaved roads whole day. Cr(VI) has been linked to coal and gasoline combustion in vehicle operations (Leili et al. 2008). The US-EPA sets the limit for airborne Cr(VI) at 12 ng m^−3^ (USEPA 1999); however, all monitoring sites exceeded this value by a factor of 5.5– 8.7 in the three zones. The Cr(VI) levels linked to PM_10_ are significantly superior to the results shown in previous studies (Cheng et al. 2017), and the *I*_*geo*_ confirms the presence of anthropogenic activities (Fig. 2). For example, in a coal mine in the arid desert region of northwest China, Cr(VI) levels during winter 2019 were 0.02 ± 0.02 µg m^−3^ (Liu et al. 2022). Roy et al. (2019b) determined the PM_10_-bound metal concentrations in one of the India's largest coalfields. The authors found mean concentrations of Al, Cr(VI), Mn, Cu, Pb, and Zn of 18.77 ± 12.39 µg m^−3^, 0.06 ± 0.02 µg m^−3^, 1.64 ± 0.66 µg m^−3^, 6.32 ± 3.17 µg m^−3^, 0.86 ± 0.51 µg m^−3^, and 1.40 ± 0.80 µg m^−3^, respectively. These values are higher than the present results, except for Cr(VI).

PAH concentrations do not exceed the threshold limit values established by different environmental agencies (ACGIH 2005; ATSDR 2023). BaP is widely acknowledged as the most toxic PAH, and its toxicological characteristics have been extensively studied (Agudelo-Castañeda et al. 2017; Feretti et al. 2019). Daily average BaP concentrations were found to range between 0.41 and 0.60 ng m^−3^ with a maximum value of 0.92 ng m^−3^ (Table 3). This means that the permissible limit (1 ng m^−3^) was not exceeded (Baan et al. 2009; IARC 2012). These concentrations are lower than those found in PM-bound BaP from other open-pit coal mines (Roy et al. 2019a; Zheng et al. 2019). The ratio of different PAHs allows us the identification of their potential emission sources. The Ant/(Ant + Phe) ratio > 0.10 indicate a robust pyrogenic emission source, while the Ant/(Ant + Phe) ratio < 0.10 suggests petrogenic emission sources (Suman et al. 2016). The FR/(FR + Pyr) ratio > 0.60 is indicative of biomass burning, fuel oil, and coal combustion sources (Yunker et al. 2002). On the other hand, the BbF/(BbF + BkF) ratio > 0.50 would imply that diesel combustion plays a vital role as an emissions source (Yang et al. 2020). For every zone, PAH ratios were calculated (Table S5). It was found that the PAHs are mainly associated with coal combustion, vehicle emissions, and biomass burning. The mean BbF/(BbF + BkF) ratio (95th percentile value) was in the range of 0.47–0.55 (0.62–0.78), suggesting that vehicular emissions play an essential role in ambient PM_10_. The mean Ant/(Ant + Phe) ratio was above 0.97 in the whole study area. These pyrogenic sources are related to the incomplete combustion of biomass or biofuels. The mean RF/(FR + Pyr) ratio of 0.98 for the three zones confirms significant coal combustion and biomass burning emissions.

*HI* values were above considered safe values (1.0) with averages of 6.10, 3.32, and 3.61 for the North Zone, South Zone, and Populated Zone, respectively (Fig. 5). Several previous studies conducted in opencast coal mines have indicated that the *HQ* values for Cr(VI) exceed the estimated values in the South Zone (0.64) and Populated Zone (0.66), but fall below the estimated values in the North Zone of the present study (1.06) (Liu et al. 2022; Zazouli et al. 2021). In open-pit mining complexes larger than our study domain, *HQ* values via inhalation were reported to be lower than reported in our results. For example, the *HQ* values for Cr(VI) and Cu ranged from 0.03 –0.10 and 0.01 –0.10, respectively, in Jharia (India). This city is considered one of the most important coalfields in India. However, the *HQ* values for Mn levels were much higher, ranging from 3.55 –22.70, surpassing our findings in the same area (Roy et al. 2019b). Jena and Singh (2016) calculated the potential health risks (non-cancer and carcinogenic) associated with different trace elements (As, Cd, Co, Cr(VI), Cu, Fe, Mn, Ni, Pb, and Zn) in PM_10_ at a mining site in Dhanbad (known as the coal capital of India). The authors estimated the *HI* values of 1.30 for exposure in adults in mining areas, where Cr(VI) contributes with 91% of the estimated total risk.

The mean values of As (2.58 × 10^–05^ –8.68 × 10^–05^) and Pb (5.64 × 10^–08^ –1.37 × 10^–07^) are considered within the acceptable risk ranges. On the other hand, the average range of CR for Cr(VI) was above 3.30 × 10^–04^, a value that exceed the limits of tolerable risk (Table 4). Emission sources play a significant role in PM-bound Cr(VI) in the vicinity and downwind of mining activities. In a desert region of northwest China where thermal coal storage and processing activities are carried out, the *CR* of Cr(VI) due to inhalation of PM was higher in spring than in winter. The *CR* of Cr(VI) in adult males (2.57 × 10^–03^) was the highest compared to the other PM-associated metals (Liu et al. 2022). Heavy metals bound to PM_10_ were measured in a Coalfield industrial complex in Singrauli (India), for two consecutive years (2016 and 2017). The author estimated that the *CR* associated with As, Cd, Cr(VI), and Ni exposure in PM_10_ ranged from 6.66 × 10^–12^ and 3.31 × 10^–08^ (Yadav 2021). On the other hand, in Dhanbad (India), a city influenced by mining activities, high *CR* from Cr(VI) in PM_10_ were 1.03 × 10^–04^ and 5.12 × 10^–03^ in adults and children, respectively. These results demonstrate the importance of identifying the sources of Cr(VI) emissions and selecting population groups (adults or children) to reduce uncertainty associated health risk assessment. On the other hand, the *ILCR* determined that exposure and inhalation of PAHs were acceptable in the domain zone. BaP contributed 54% –56% of the total inhalation risk of PM10-bound PAHs, while IDP contributed 16%–19%. Risk assessment for PM_10_-bound PAHs exposure in other coal mines has found *ILCR* higher than the present results. For example, Roy et al. (2019a) quantified the *CR* in the main mining area in the Dhanbad District (India) using estimates of PM_10_-bound PAHs. The authors found the *CR* of PAH inhalation exposure in the range of 1.45 × 10^–06^ –0.53 × 10^–05^ during 2012–2013 at six sites influenced by roadway emissions, vehicular traffic, overburden removal, blasting, mineral haulage, mechanical handling operations, stockpiles, and site restoration. In Candiota city (Brazil), an *ILCR* value of 2.8 × 10^–06^ (reference values of US-EPA) and 2.6 × 10^–06^ (real values of Candiota`s inhabitants) was found during a monitoring campaign from July 2012 to March 2013 (Marmett et al. 2023).

The CPF analysis indicated that contributions from external sources and mining activities were predominant when winds were blowing from the east. Moreover, given the predominant wind direction, the estimated risk levels reach a significant radius of more than 30 km from the center of the mining complex. This study possesses a unique approach in utilizing the CPF method to acquire the spatial distribution of HI, CR, and ILCR. CPF method, in conjunction with forward-back-trajectory cluster analysis, provides a deeper understanding of the sources and risk areas involved. Compared to other spatial risk analyses, the CPF method is more effective in evaluating the coal mines' atmospheric environment capacity. It can capture a more comprehensive range of information about the distributions of atmospheric pollutants and emission sources, which is impossible with the existing geographical risk distribution method that relies on concentration–response relationship estimation, data extrapolation, and statistical models based on environmental variables. Typically, the current approach considers the target emission sources and external sources proportionally in time and space, contributing to the PM_10_ concentrations (Sun et al. 2019). However, not including external sources could lead to a loss of information as they cannot account for the accumulated impact of pollutants. To address this, the CPF method introduced in this study synthesizes the effects of various sources by converting them into health risks. Consequently, all the impacts of the sources can be retrieved.

Although we assessed the potential health risks via inhalation by heavy metals and PAHs linked to PM_10_ at the largest open-pit coal mine in Latin America, our study has some inherent limitations. We collected PM_10_ samples from February to May to evaluate the potential risks (carcinogen and non-carcinogen). The sampling period may not represent exposure concentrations spanning a lifetime. Another limitation is that the PM_10_ exposure concentration for each person is based on the generalized assumption of ambient PM_10_ concentration. However, despite the constraints and associated uncertainties, our study shows evidence of the impact of PM_10_-bound heavy metals and PAHs on health in the study area. Furthermore, the obtained results were achieved using scientifically rigorous methods. Therefore, it can be helpful in the establishment of policies and regulation-control of emission sources to achieve sustainable development goals.

## Conclusions

This study evaluated the potential risk associated with heavy metals and PAHs linked in PM_10_ in three zones of the largest Latin America's open-pit mining area. The highest concentrations of PM_10_-bound heavy metals were detected for Al and Cr(VI). PM_10_-bound Al daily average was 1420.90 ng m^−3^ and 1658.94 ng m^−3^, while PM_10_-bound Cr(VI) had daily averages of 57.51 ng m^−3^ and 104.16 ng m^−3^ for the three zones. The average total concentration of the 14 PAHs was 8.39 ± 0.15 ng m^−3^, 10.40 ± 0.17 ng m^−3^, and 7.21 ± 0.16 ng m^−3^ for the North Zone, South Zone, and Populated Zone, respectively. In our study, the average range of *CR* for heavy metals was 3.30 × 10^–04^ –5.47 × 10^–04^, a range that exceed the limits of tolerable risk. On the contrary, the cancer risk due to PAH exposure was acceptable, with mean *ILCR* values calculated to vary between 2.87 × 10^–07^ and 4.21 × 10^–07^. The risks estimated in the population around the open-cast coal mine warn about the harmful consequences of the levels of heavy metals linked to PM_10_, especially the Cr(VI) levels. Thus, it is advisable to establish control on PM_10_ emissions due to sources such as coal combustion, biomass burning, and vehicles emission. These findings underscore the need for comprehensive measures to address the environmental impact of coal mining and protect the health and wellbeing of individuals and communities living in these regions. Additionally, specific public policies must be established in this region that seek greater control over the emissions sources.

### Supplementary Information

Below is the link to the electronic supplementary material.

Supplementary file1 (DOCX 308 KB)

## Data Availability

The data underlying this article will be shared on reasonable request to the corresponding author.

## Nomenclature

PM: Particulate matter
PAHs: Polycyclic Aromatic Hydrocarbons
I_geo: Geo-accumulation index
CR: Lifetime excess cancer risk linked to heavy metal
ILCR: Incremental lifetime cancer risk linked to PAHs
CPF: Conditional probability function method
