# Supplementary material for: Human health risk assessment of PM10-bound heavy metals and PAHs around the Latin America’s Largest opencast coal mine
Source: Environ Sci Pollut Res Int. 2023 Nov 27;30(60):125915–30. doi: 10.1007/s11356-023-30787-z (PMC10754741; doi:10.1007/s11356-023-30787-z)
Supplement: Supplementary file 1 — Supplementary file1 (DOCX 308 KB) [file 11356_2023_30787_MOESM1_ESM.docx]

Human health risk assessment of PM_10_-bound heavy metals and PAHs around the Latin America’s Largest opencast coal mine

Heli A. Arregocés^1, 2*^, Guillermo J. Bonivento^1,3^, Luis A. Ladino^4^, E. Beristain-Montiel^5^, Gloria Restrepo^2^, Javier Miranda^6^, Harry Alvarez-Ospina^7^, Roberto Rojano^1^

^1^Grupo de Investigación GISA, Facultad de Ingeniería, Universidad de La Guajira, Riohacha, Colombia

^2^Grupo Procesos Fisicoquímicos Aplicados, Facultad de Ingeniería, Universidad de Antioquia SIU/UdeA, Calle 70 No. 52–21, Medellín, Colombia

^3^Grupo de Investigación ZENTECH, Facultad de Ingeniería, Pontificia Universidad Javeriana, Cra 7 No. 40–62, Bogotá, Colombia

^4^Instituto de Ciencias de la Atmósfera y Cambio Climático, Universidad Nacional Autónoma de México, México City, México

^5^Facultad de Química, Universidad Nacional Autónoma de México, México City, México

^6^Instituto de Física, Universidad Nacional Autónoma de México, México City, México

^7^Facultad de Ciencias, Universidad Nacional Autónoma de México, México City, México

^*^ Corresponding author. Tel: +57-035-728-0206

E*-mail address*: [harregoces@uniguajira.edu.co](mailto:harregoces@uniguajira.edu.co)

**Observed and Predicted** **surface meteorological variables**

In the supplementary Figure S2, we compare the daily T, RH, and WS values for the ZS_02 site within the domain site between the simulated and observed data. All the target variables had good correspondence between simulated and observed values (r>0.63). Daily T and RH values tend to be underestimated by the WRF model, while daily WS values reached a Bias of 0.52 m s^-1^.

| 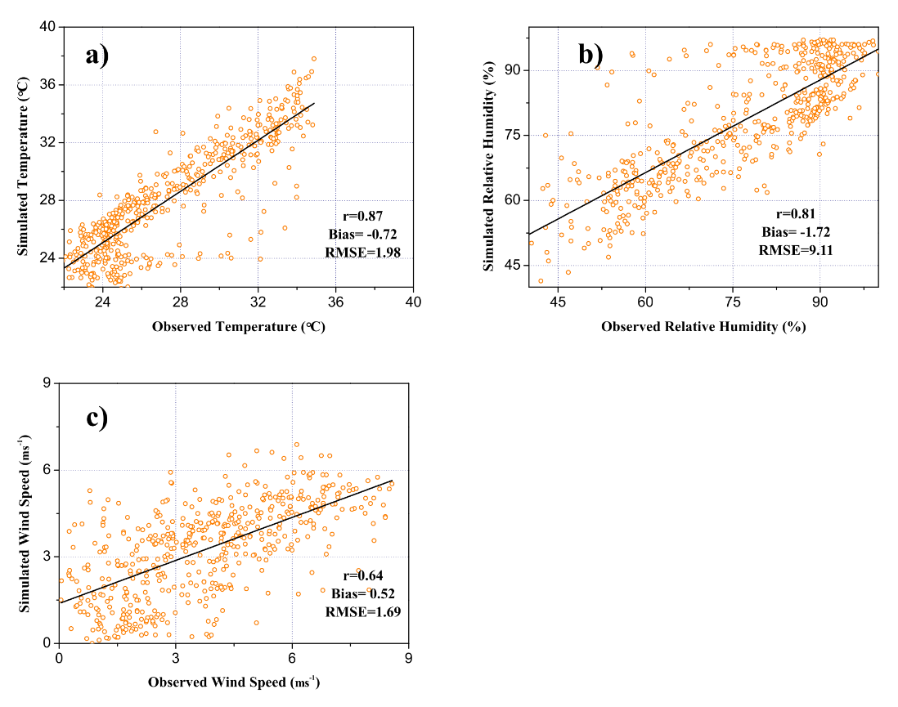 |
| --- |
| Figure S1. Observed and WRF simulated surface meteorological variables (a) Surface air temperature at 2 m (T), (b) Relative Humidity at 2 m (RH), and (c) Surface wind speed at 10 m (WS). |

Table S1. Percentage of uncertainty for the samples according to each study area

|  | North Zone | South Zone | Populated Zone |
| --- | --- | --- | --- |
| Al | 6.5% | 6.5% | 6.5% |
| Cr | 6.5% | 6.6% | 6.6% |
| Mn | 8.2% | 9.2% | 8.9% |
| Cu | 7.3% | 7.5% | 7.9% |
| Zn | 6.6% | 6.5% | 6.6% |
| As | 9.2% | 8.9% | 8.2% |
| Pb | 7.9% | 13.6% | 8.2% |

Table S2. Selected ion masses and recovery of PAHs.

| PAHs | Abbreviations | Selected ion masses (u) | Recovery (%) |
| --- | --- | --- | --- |
| Naphtalene | Nap | 128 | 72 |
| Acenaphtylene | Acy | 152 | 68 |
| Acenaphthene | Ace | 154 | 68 |
| Fluorene | Flu | 166 | 87 |
| Phenanthrene | Phe | 178 | 68 |
| Anthracene | Ant | 178 | 83 |
| Fluoranthene | FR | 202 | 75 |
| Pyrene | Pyr | 202 | 78 |
| benzo[a]anthracene | BaA | 228 | 87 |
| Triphenylene | Tri | 228 | 87 |
| Indeno[1,2,3-cd]pyrene | IDP | 276 | 61 |
| Benzo[b]fluoranthene | BbF | 252 | 69 |
| Benzo[a]pyrene | BaP | 252 | 70 |
| Benzo[k]fluoranthene | BkF | 252 | 68 |

Table S3. Human health risks parameters of PM_10_-bound heavy metals and PAHs

| Factors | Notation | Unit | Values |
| --- | --- | --- | --- |
| Exposure frequency | $EF$ | Days years^-1^ | 365 |
| Exposure Duration | $ED$ | year | 30 |
| Exposure time | $ET$ | h day^-1^ | 24 |
| Inhalation rate | $IR$ | m^3^ day^-1^ | 20 |
| Body weight | $BW$ | kg | 70 |
| Average lifetime | ${AT}_{n}$ | hours | ED×365×24 (non-carcinogens)  70×365×24 (Carcinogens) |
| Chronic inhalation reference concentrations | ${RfC}_{i}$ | mg m^-3^ | Al (5× 10^-3^), Cr (1× 10^-4^), Mn (5× 10^-5^), Fe, Cu (2× 10^-5^), Zn (9× 10^-4^), As (2× 10^-5^), and Pb (5× 10^-4^) |
| Conversion factor | $CF$ | μg mg^-1^ | 1000 |
| Inhalation Unit Risk | $IUR$ | (μg m^-3^)^-1^ | Cr (1.20× 10^-2^), As (4.30× 10^-3^), Pb (1.20× 10^-5^) |
| Toxicity equivalency factor | ${TEF}_{i}$ | ng m^-3^ | Nap (0.001), Acy (0.001), Ace (0.001), Flu (0.001), Phe (0.001), Ant (0.01), FR (0.05), Pyr (0.001), BaA (0.1), IDP (0.07), BbF (0.1), BaP (1) and  BkF (0.1) |
| Inhalation carcinogenic slope factor | $SF$ | mg kg^-1^day^-1^ | 3.14 (Peng et al., 2011) |
| Average lifespan | $AT$ | days | 70×365 |

Table S4. Parametrization and performance statistics of the WRF model during simulations over a Latin America’s Largest opencast coal mine area from February 1 to May 31, 2022.

| Parameters | Values |
| --- | --- |
| Dynamics | Non-hydrostatic |
| Resolution horizontal | Domain 1: 27 × 27 km, Domain 2: 9 × 9 km, Domain 3: 3 × 3 km, Domain 4: 1 × 1 km |
| ref_lat, ref_lon | 11.4, -72.5 |
| Vertical resolution | The vertical resolution comprises 60 levels ( = 1.000, 0.993, 0.991, 0.989, 0.986, 0.982, 0.978, 0.975, 0.973, 0.972, 0.971, 0.967, 0.962, 0.960, 0.958, 0.949, 0.942, 0.938, 0.936, 0.927, 0.921, 0.917, 0.913, 0.910, 0.902, 0.895, 0.884, 0.873, 0.865, 0.861, 0.854, 0.851, 0.832, 0.821, 0.765, 0.719, 0.672, 0.622, 0.571, 0.520, 0.468, 0.442, 0.420, 0.376, 0.335, 0.298, 0.282, 0.263, 0.255, 0.231, 0.202, 0.185, 0.175, 0.150, 0.146, 0.127, 0.106, 0.088, 0.055, 0.040, 0.020) |
| Surface Layer  Land Surface | Similarity Scheme (Janjic, 2002)  Unified Noah Land Surface Model (Tewari et al., 2004) |
| Microphysics scheme  PBL schemes | WRF Single–moment 3–class (Hong et al., 2004) Eta Mellor–Yamada–Janjic Scheme (MYJ) (Janjic, 1994) |
| BIAS | Temperature at 2 meter (-0.03 °C), Relative humidity (-0.02 %),  Wind speed (0.16 m s^-1^) |
| Mean absolute error | Temperature at 2 meter (1.6 °C), Relative humidity (6.8 %),  Wind speed (1.4 m s^-1^) |
| Normalized root means square error | Temperature at 2 meter (0.1), Relative humidity (0.1),  Wind speed (0.6) |

Table S5. Summary statistics of PAHs ratios for identification of contamination sources.

| Ratio | North Zone | | | | South Zone | | | | Populated Zone | | | |
| --- | --- | --- | --- | --- | --- | --- | --- | --- | --- | --- | --- | --- |
|  | Mean | SD | 5th | 95th | Mean | SD | 5th | 95th | Mean | SD | 5th | 95th |
| BbF/(BbF + BkF) | 0.47 | 0.13 | 0.33 | 0.65 | 0.55 | 0.14 | 0.38 | 0.78 | 0.49 | 0.11 | 0.35 | 0.62 |
| Ant/(Ant + Phe) | 0.90 | 0.03 | 0.85 | 0.94 | 0.88 | 0.03 | 0.84 | 0.92 | 0.91 | 0.04 | 0.83 | 0.95 |
| FR/(FR+ Pyr) | 0.98 | 0.01 | 0.97 | 0.99 | 0.98 | 0.01 | 0.97 | 0.99 | 0.98 | 0.00 | 0.97 | 0.99 |

| *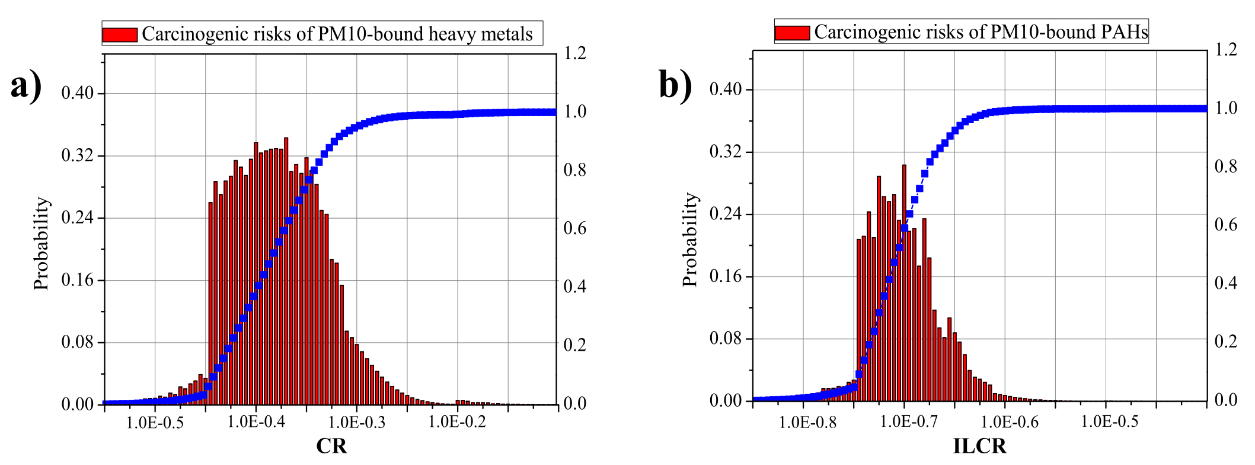* |
| --- |
| Figure S2. Probability distribution and cumulative probability of the carcinogenic risks (a) through inhalation for PM_10_-bound heavy metals (b) through inhalation for PM_10_-bound heavy metals. Using Monte Carlo simulations, risk analysis was conducted based on the targeted metals and PAHs' mean concentrations. |
